# Genetic association between *TNF-α* promoter polymorphism and susceptibility to squamous cell carcinoma, basal cell carcinoma, and melanoma: A meta-analysis

**DOI:** 10.18632/oncotarget.17179

**Published:** 2017-04-18

**Authors:** Ning Liu, Guang-Jing Liu, Juan Liu

**Affiliations:** ^1^ Department of Plastic and Burns Surgery, The First Center Hospital of Tianjin, Tianjin 300192, PR China; ^2^ Department of General Surgery, Bailou Hospital, Tianjin 300040, PR China

**Keywords:** TNF-α, single nucleotide polymorphism, SCC, BCC, melanoma

## Abstract

Tumor necrosis factor-alpha (TNF-α) is a multifunctional pro-inflammatory cytokine that plays an important role in cancer development. We performed a meta-analysis to assess the relationship between single nucleotide polymorphisms in the TNF-α promoter region (rs1800629 and rs361525) and susceptibility to squamous cell carcinoma (SCC), basal cell carcinoma (BCC) and melanoma. After database retrieval, article selection, data extraction, and quality assessment, 20 articles comprising 4865 cases and 6329 controls were included in this study. rs1800629 was associated with an increased overall risk of SCC, lung SCC, and oral SCC in the AA vs G and AA vs GG+GA genetic models (all OR>1, *P_association_*<0.05). No increased risk of skin SCC, skin BCC or melanoma was observed (all *P_association_*>0.05). Rs361525 was not associated with overall SCC risk in the allele, heterozygote, dominant, recessive, or carrier model (all *P_association_*>0.05). Begg's and Egger's tests (*P*_Begg_>0.05; *P*_Egger_>0.05) demonstrated there was no significant publication bias. These data indicate that the AA genotype of *TNF-α* rs1800629, but not rs361525, is associated with an increased risk of SCC, suggesting it could potentially serve as a prognostic marker for predicting SCC risk.

## INTRODUCTION

Tumor necrosis factor-alpha (*TNF-α*) gene, located on chromosome 6p21.231, consists of four exons [1, 2]. TNF-α protein, encoded by *TNF-α* gene, is associated with cellular differentiation, proliferation, apoptosis, inflammatory responses, insulin resistance, and tumorigenesis [2–4]. Several single nucleotide polymorphisms (SNPs), including rs1800629 (−308 G/A) and rs361525 (−238 G/A), have been identified in the promoter region of *TNF-α* gene [2].

The role of *TNF-α* gene mutations in the risk of squamous cell carcinoma (SCC) remains inconclusive. For instance, the rs1800629 polymorphism of *TNF-α* gene has been linked to the risk of esophageal SCC in northern Indian patients [5], but not in Kazakh patients [6]. *TNF-α* rs1800629 polymorphism has been associated with the risks of oral SCC in Taiwan [7], but not in northern Indian population, which has been linked with rs361525 polymorphism [8]. There was also no association between the rs1800629 polymorphism and lung SCC risk in the German population [9].

Skin cancer comprises cutaneous melanoma, skin SCC (SSCC), and skin basal cell carcinoma (SBCC) [10]. Allelic variants of *TNF-α* gene have been reported to contribute to the risk of skin cancer in certain populations. For example, the study by Rizzato et al. has indicated that *TNF-α* rs1800629 might affect the SBCC risk in Caucasian population [11]. The A allele or GA genotype of *TNF-α* gene rs1800629 polymorphism was also reported to influence the course of BCC in Polish population [12]. However, the role of *TNF-α* polymorphisms in skin cancer is still inconclusive. For example, Skov et al. reported that TNF-α release, but not rs1800629 polymorphism, was linked to the SBCC risk in Caucasian population [13]. To our knowledge, no meta-analysis has been previously performed to assess the link between *TNF-α* polymorphisms and the risk of skin cancer.

Therefore, in this study, we carried out a comprehensive systematic review and meta-analysis to determine the association of *TNF-α* polymorphisms and the risk of skin cancer and different SCC diseases.

## RESULTS

### Characteristics of studies included in meta-analysis

**Figure 1:**
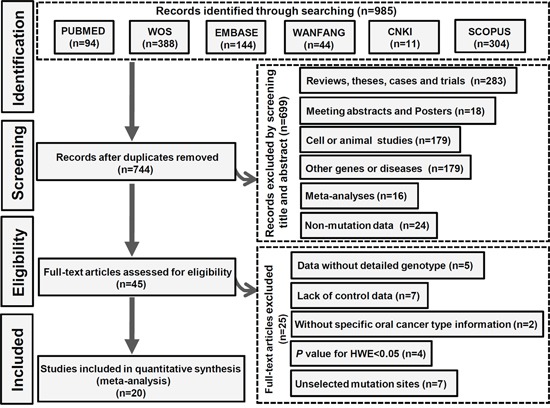
The selection process of the meta-analysis

Six databases, including PUBMED, Web of Science (WOS), EMBASE, WANFANG, CNKI, and SCOPUS, were electronically searched on January 17^th^, 2017 to identify the eligible studies. The search details are shown in Supplementary Table 1. Flowchart of the search strategy and article selection for meta-analysis is shown in Figure 1. Briefly, 985 related articles were obtained from the above databases. After 241 duplicated articles were removed, 699 articles were excluded by screening the title and abstract. The eligibility of 45 full-text articles was then assessed, and 25 articles were excluded. The results are shown in Supplementary Table 2. Finally, 20 eligible articles with 4865 cases and 6329 controls were included for quantitative synthesis [1, 5–9, 11–24]. All selected articles met the inclusion and exclusion criteria. We used the Newcastle-Ottawa Scale (NOS) to assess the quality of the studies. As shown in Supplementary Table 3, the NOS scores of all studies were equal to or greater than 7, indicating a high quality. After covariate adjustment in logistic regression, the characteristics and genotype distributions of included studies are shown in Tables 1 and 2.

**Table 1:** Characteristics of studies included in meta-analysis

First author[Ref]	Year	Country	Ethnicity	Number		Source of controls	Age (mean value)		Genotyping assay	Gender (male %)	
				Case	Control		Case	Control		Case	Control
Cui [6]	2015	China	Asian	212	200	Population	52.5	54.4	PCR-RFLP	59.7%	51.8%
Flego [14]	2009	Croatia	Caucasian	113	230	Population	NA	NA	PCR-RFLP	NA	NA
Gu [15]	2009	USA	Caucasian	212	211	Population	NA	NA	ARMS-PCR	NA	NA
Gupta [1]	2008	India	Asian	94	133	Population	50.7	NA	PCR-RFLP	80.9%	NA
Huang [19]	2005	China	Asian	65	65	Population	65.0	55.0	PCR-RFLP	81.5%	77.0%
Kietthubthew [24]	2010	Thailand	Asian	97	152	Population	67.5	69.7	Taqman PCR	73.8%	55.4%
Kostic [17]	2013	Serbia	Caucasian	50^#^	60	Population	69.0^#^	NA	PCR-RFLP	70.0%^#^	NA
				50^&^	60	Population	73.0^&^	NA	PCR-RFLP	40.0%^&^	NA
Liu [7]	2005	China	Asian	192	146	Population	54.1	52.0	PCR-RFLP	90.1%	89.0%
Oh [23]	2010	USA	Caucasian	75	839	Population	NA	NA	SNPlex assay	NA	NA
Rizzato [11]	2011	Mixed	Caucasian	506	515	Hospital	67.0	61.0	KASPar SNP genotyping	44.8%	51.4%
Seifart [9]	2005	Germany	Caucasian	40	242	Population	65.4	37.9	PCR-RFLP	NA	55.1%
Shih [20]	2006	China	Asian	83	205	Population	NA	62.8	PCR-RFLP+ sequencing	NA	66.3%
Singh [8]	2015	India	Asian	272	185	Population	47.7	43.1	PCR-RFLP	80.5%	76.8%
Skov [13]	2003	Denmark	Caucasian	191	107	Population	65.9	64.6	PCR-RFLP	59.2%	53.3%
Sobjanek [12]	2015	Poland	Caucasian	176	261	Population	68.9	NA	ARMS-PCR	46.6%	NA
Umar [5]	2013	India	Asian	290	311	Population	57.0	55.0	ARMS-PCR	72.8%	71.1%
Welsh [16]	2011	USA	Caucasian	894^&^	816	Population	58.7^&^	61.3	Taqman PCR	56.0%^&^	59.9%
				681*	816	Population	64.1*	61.3	Taqman PCR	63.5%*	59.9%
Whiteman [18]	2010	Australia	Caucasian	207	1293	Population	NA	NA	Sequenom iPLEX	58.0%	66.0%
Yang [21]	2011	China	Asian	205	198	Population	49.3	48.9	Taqman PCR	100.0%	100.0%
Zhang [22]	2011	China	Asian	160	160	NA	NA	NA	PCR-SSP	NA	NA

Ref: reference; #: OSCC group; &: SBCC group; *: SSCC group; NA: not available; PCR-RFLP: polymerase chain reaction-restriction fragment length polymorphism; ARMS-PCR: amplification refractory mutation system-polymerase chain reaction; Taqman PCR: Taqman polymerase chain reaction; PCR-SSP: Polymerase chain reaction-sequence specific primer.

**Table 2:** The genotype data of studies included in meta-analysis

Group	SNP	First author [Ref]	Year	Case				Disease	Control				HWE	
				Total	G/G	G/A	A/A		Total	G/G	G/A	A/A	χ^2^	*P*
**SSC**	rs1800629	Flego [14]	2009	113	79	30	4	LSCC	230	171	53	6	0.59	0.44
		Huang [19]	2005	65	64	1	0	LSCC	65	55	10	0	0.45	0.50
		Oh [23]	2010	75	56	18	1	LSCC	839	632	194	13	0.19	0.67
		Seifart [9]	2005	40	24	15	1	LSCC	242	171	67	4	0.79	0.37
		Shih [20]	2006	83	50	25	8	LSCC	205	169	34	2	0.04	0.84
		Gupta [1]	2008	94	61	23	10	OSCC	133	114	19	0	0.79	0.38
		Kietthubthew [24]	2010	97	83	14^&^	-	OSCC	152	133	19^&^	-	NA	>0.05
		Kostic [17]	2013	50	35	14	1	OSCC	60	39	21	0	2.70	0.10
		Liu [7]	2005	192	175	16	1	OSCC	146	120	24	2	0.39	0.53
		Singh [8]	2015	272	235	35	2	OSCC	185	164	20	1	0.21	0.65
		Cui [6]	2015	212	150	57	5	ESCC	200	140	58	2	2.29	0.13
		Umar [5]	2013	290	227	62	1	ESCC	311	268	42	1	0.23	0.63
		Whiteman [18]	2010	207	128	71	8	ESCC	1293	842	403	48	0.00	0.98
		Zhang [22]	2011	160	135	23	2	ESCC	160	140	18	2	2.36	0.12
		Yang [21]	2011	205	180	23	2	OPSCC	198	155	43	0	2.94	0.09
		Welsh [16]	2011	681	476	188	17	SSCC	816	571	223	22	0.00	0.97
**SSC**	rs361525	Kietthubthew [24]	2010	97	92	5^&^	-	OSCC	152	141	11^&^	-	NA	>0.05
		Liu [7]	2005	192	188	4	0	OSCC	146	136	10	0	0.18	0.67
		Singh [8]	2015	272	252	20	0	OSCC	185	180	5	0	0.03	0.85
		Flego [14]	2009	113	108	5	0	LSCC	230	214	16	0	0.30	0.58
		Shih [20]	2006	83	75	8	0	LSCC	205	161	44	0	2.96	0.09
		Yang [21]	2011	205	200	5	0	OPSCC	198	187	11	0	0.16	0.69
**skin cancer**	rs1800629	Gu [15]	2009	212	156	46	10	melanoma	211	140	61	10	0.98	0.32
		Kostic [17]	2013	50	29	21	0	SBCC	60	39	21	0	2.70	0.10
		Rizzato [11]	2011	506	358	128	20	SBCC	515	390	117	8	0.05	0.82
		Skov [13]	2003	191	133	49	9	SBCC	107	68	37	2	1.45	0.23
		Sobjanek [12]	2015	176	134	41	1	SBCC	261	178	80	3	3.38	0.07
		Welsh [16]	2011	894	612	265	17	SBCC	816	571	223	22	0.00	0.97
				681	476	188	17	SSCC	816	571	223	22	0.00	0.97

Ref: reference; SCC: squamous cell carcinoma; SNP: single nucleotide polymorphisms; &: the number of GA+AA; NA: not available; LSCC: lung squamous cell carcinoma; OSCC: oral squamous cell carcinoma; ESCC: esophageal squamous cell carcinoma; OPSCC: oral and pharyngeal squamous cell carcinoma; SSCC: skin squamous cell carcinoma; SBCC: skin basal cell carcinomas; HWE: Hardy-Weinberg equilibrium.

### Association between *TNF-α* rs1800629 polymorphism and the risk of SCC

Meta-analysis of 16 studies [1, 5–9, 14, 16–24] comprising 2836 cases and 5235 controls was performed to analyze the association between *TNF-α* rs1800629 polymorphism and the risk of SCC under allele model (A vs G), homozygote model (AA vs GG), heterozygote model (GA vs GG), dominant model (GA+AA vs GG), recessive model (AA vs GG+GA), and carrier model (carrier A vs G). Pooled analysis data are shown in Table 3. Compared with the control group, no significant overall SCC risk was observed in the case group under A vs G model (OR=1.18, 95% CI=0.92∼1.51, *P_association_*=0.192), GA vs GG model (OR=1.10, 95% CI=0.87∼1.39, *P_association_*=0.439), GA+AA vs GG (OR=1.15, 95% CI=0.90∼1.47, *P_association_*=0.255), or carrier A vs G model (OR=1.12, 95% CI=0.91∼1.39, *P_association_*=0.287). However, an increased overall SCC risk was observed in AA vs GG model (OR=1.62, 95% CI=1.15∼2.29, *P_association_*=0.006) and AA vs GG+GA model (OR=1.56, 95% CI=1.10∼2.20, *P_association_*=0.012).

**Table 3:** Pooled analysis for the association between *TNF-α* rs1800629 polymorphism and the risk of SCC

Comparison	Subgroup	Test of association				Number		
		ORs	95% CIs	z	*P_association_*	Studies	Case	Control
**A vs G**	overall	1.18	0.92∼1.51	1.30	0.192	15	2739	5083
	Asian	1.18	0.73∼1.92	0.67	0.501	9	1573	1603
	Caucasian	1.07	0.93∼1.22	0.94	0.349	6	1166	3480
	ESCC	1.19	0.99∼1.44	1.84	0.066	4	869	1964
	LSCC	1.39	0.72∼2.36	0.87	0.385	5	376	1581
	OSCC	1.19	0.49∼2.88	0.39	0.697	4	608	524
**AA vs GG**	overall	1.62	1.15∼2.29	2.73	**0.006**	14	2674	5018
	Asian	3.67	1.89∼7.16	3.82	**<0.001**	8	1508	1538
	Caucasian	1.08	0.70∼1.66	0.33	0.743	6	1166	3480
	ESCC	1.24	0.66∼2.32	0.68	0.497	4	869	1964
	LSCC	2.72	1.32∼5.61	2.72	**0.007**	4	311	1516
	OSCC	3.91	1.38∼11.05	2.57	**0.010**	4	608	524
**GA vs GG**	overall	1.10	0.87∼1.39	0.77	0.439	15	2739	5083
	Asian	1.05	0.67∼1.64	0.20	0.839	9	1573	1603
	Caucasian	1.08	0.92∼1.27	0.97	0.334	6	1166	3480
	ESCC	1.23	0.95∼1.61	1.55	0.120	4	869	1964
	LSCC	1.27	0.74∼2.18	0.85	0.393	5	376	1581
	OSCC	0.99	0.51∼1.95	0.02	0.986	4	608	524
**GA+AA vs GG**	overall	1.15	0.90∼1.47	1.14	0.255	16	2836	5235
	Asian	1.14	0.73∼1.77	0.56	0.577	10	1670	1755
	Caucasian	1.08	0.93∼1.26	0.99	0.324	6	1166	3480
	ESCC	1.24	0.97∼1.57	1.73	0.084	4	869	1964
	LSCC	1.31	0.71∼2.40	0.86	0.389	5	376	1581
	OSCC	1.12	0.58∼2.15	0.33	0.738	5	705	676
**AA vs GG+GA**	overall	1.56	1.10∼2.20	2.53	**0.012**	14	2674	5018
	Asian	3.52	1.80∼6.88	3.68	**<0.001**	8	1508	1538
	Caucasian	1.05	0.68∼1.62	0.22	0.829	6	1166	3480
	ESCC	1.19	0.64∼2.22	0.56	0.577	4	869	1964
	LSCC	2.48	1.20∼5.12	2.45	**0.014**	4	311	1516
	OSCC	3.84	1.34∼11.01	2.50	**0.012**	4	608	524
**carrier A vs G**	overall	1.12	0.91∼1.39	1.06	0.287	15	2739	5083
	Asian	1.12	0.74∼1.69	0.52	0.604	9	1573	1603
	Caucasian	1.06	0.91∼1.22	0.73	0.468	6	1166	3480
	ESCC	1.17	0.96∼1.42	1.59	0.111	4	869	1964
	LSCC	1.24	0.75∼2.07	0.84	0.402	5	376	1581
	OSCC	1.10	0.54∼2.24	0.27	0.789	4	608	524

ESCC: esophageal squamous cell carcinoma; LSCC: lung squamous cell carcinoma; OSCC: oral squamous cell carcinoma; Ors: odd ratios; CIs: confidence intervals.

**Figure 2:**
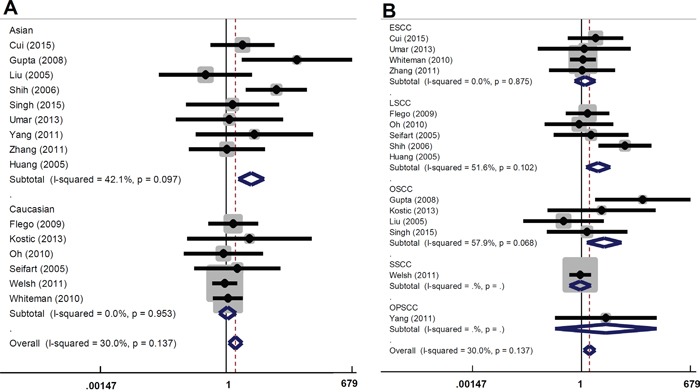
Forest plot for the association between *TNF-α* rs1800629 polymorphism and the risk of SCC under AA vs GG model **(A)** Subgroup analyses based on ethnicity; **(B)** Subgroup analyses based on disease type.

**Figure 3:**
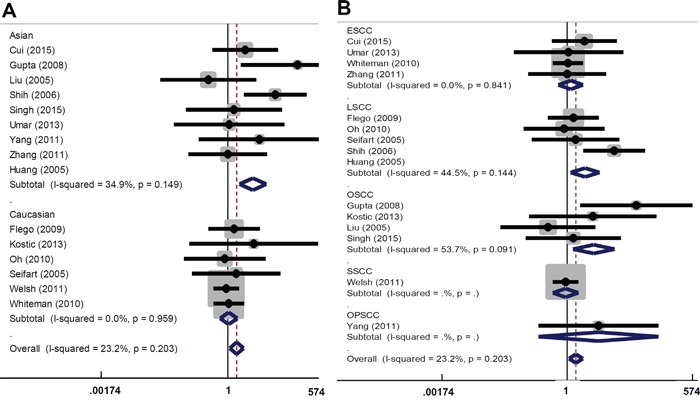
Forest plot for the association between *TNF-α* rs1800629 polymorphism and the risk of SCC under AA vs GG+GA model **(A)** Subgroup analyses based on ethnicity; **(B)** Subgroup analyses based on disease type.

There are several types of SCC, including skin SCC (SSCC), esophageal SCC (ESCC), oral SCC (OSCC), and lung SCC (LSCC). We performed subgroup analyses of the above SCC types and different ethnicities under all models. As shown in Table 3 and Figure 2A, an increased overall SCC risk was observed in the Asian population under AA vs GG model (OR=3.67, 95% CI=1.89∼7.16, *P_association_*<0.001). The increased risk of LSCC (OR=2.72, 95% CI=1.32∼5.61, *P_association_*=0.007) and OSCC (OR=3.91, 95% CI=1.38∼11.05, *P_association_*=0.010) was also observed (Table 3 and Figure 2B). Similar results were observed for the AA vs GG+GA model (Table 3 and Figure 3). No significant difference was found under other genetic models (all *P_association_*>0.05). These data indicate that the AA genotype of *TNF-α* rs1800629 polymorphism correlates with the higher susceptibility towards SCC.

### Association between *TNF-α* rs361525 polymorphism and the risk of SCC

Meta-analysis of the relationship between *TNF-α* rs361525 polymorphism and susceptibility to SCC was also performed. Six studies comprising 962 cases and 1116 controls were analyzed [7, 8, 14, 20, 21, 24]. Data of pooled analysis indicated that there was no significant difference for overall SCC risk under all genetic models (Table 4 and Supplementary Figure 1; all *P_association_*>0.05). Subgroup analysis (Table 4) indicated a decreased LSCC risk under A vs G model (OR=0.49, 95% CI=0.26∼0.90, *P_association_*=0.023), GA vs GG model (OR=0.46, 95% CI=0.25∼0.87, *P_association_*=0.018); GA+AA vs GG (OR=0.46, 95% CI=0.25∼0.87, *P_association_*=0.018), and carrier A vs G (OR=0.51, 95% CI=0.27∼0.96, *P_association_*=0.037). However, only two case-control studies comprising 196 cases and 435 controls were included in the LSCC subgroup [14, 20]. No significant difference was found for other comparisons (all *P_association_*>0.05). These data indicate that *TNF-α* rs361525 polymorphism does not contribute to the risk of SCC.

**Table 4:** Pooled analysis for the association between *TNF-α* rs361525 polymorphism and the risk of SCC

Comparison	Subgroup	Test of association				Number		
		ORs	95% CIs	z	*P_association_*	Studies	Case	Control
**A vs G**	overall	0.63	0.29∼1.35	1.19	0.235	5	865	964
	Asian	0.63	0.24∼1.68	0.93	0.354	4	752	734
	LSCC	0.49	0.26∼0.90	2.28	**0.023**	2	196	435
	OSCC	0.93	0.10∼8.36	0.06	0.949	2	464	331
**GA vs GG**	overall	0.62	0.28∼1.36	1.20	0.231	5	865	964
	Asian	0.61	0.22∼1.70	0.94	0.348	4	752	734
	LSCC	0.46	0.25∼0.87	2.38	**0.018**	2	196	435
	OSCC	0.93	0.10∼8.78	0.06	0.949	2	464	331
**GA+AA vs GG**	overall	0.93	0.10∼8.78	1.39	0.165	6	962	1116
	Asian	0.63	0.28∼1.41	1.13	0.259	5	849	886
	LSCC	0.46	0.25∼0.87	2.38	**0.018**	2	196	435
	OSCC	0.86	0.23∼3.22	0.23	0.818	3	561	483
**carrier A vs G**	overall	0.64	0.31∼1.35	1.17	0.243	5	865	964
	Asian	0.64	0.25∼1.67	0.91	0.364	4	752	734
	LSCC	0.51	0.27∼0.96	2.09	**0.037**	2	196	435
	OSCC	0.93	0.11∼7.98	0.06	0.948	2	464	331

LSCC: lung squamous cell carcinoma; OSCC: oral squamous cell carcinoma; Ors: odd ratios; CIs: confidence intervals.

### Association between *TNF-α* rs1800629 polymorphism and the risk of skin cancer

We then performed meta-analysis of the relationship between *TNF-α* rs1800629 and the risk of skin cancer, including SSCC, SBCC, and melanoma. Seven studies comprising 2710 cases and 2786 controls were included [11–13, 15–17]. Data of pooled analysis indicated no significant difference under all genetic models (all *P_association_*>0.05, Table 5 and Supplementary Figure 2). Subgroup analysis (based PB and SBCC) also showed no significant difference (Table 5). However, only one case-control study was included in the subgroup analysis of melanoma [15] and SSCC [16] (Table 5). These data suggest that *TNF-α* rs1800629 polymorphism does not have a significant correlation with the risk of skin cancer.

**Table 5:** Pooled analysis for the association between *TNF-α* rs1800629 polymorphism and the risk of skin cancer

Comparison	Subgroup	Test of association				Number		
		ORs	95% CIs	z	*P_association_*	Studies	Case	Control
**A vs G**	overall	1.01	0.91∼1.12	0.24	0.814	7	2710	2786
	Caucasian	1.01	0.91∼1.12	0.24	0.814	7	2710	2786
	PB	0.95	0.85∼1.07	0.82	0.413	6	2204	2271
	SBCC	1.05	0.93∼1.20	0.82	0.411	5	1817	1759
	melanoma	0.78	0.54∼1.11	1.39	0.164	1	212	211
	SSCC	1.00	0.82∼1.21	0.04	0.964	1	681	816
**AA vs GG**	overall	1.09	0.78∼1.54	0.52	0.603	6	2660	2726
	Caucasian	1.09	0.78∼1.54	0.52	0.603	6	2660	2726
	PB	0.88	0.60∼1.29	0.65	0.513	5	2154	2211
	SBCC	1.24	0.79∼1.95	0.95	0.340	4	1767	1699
	melanoma	0.90	0.36∼2.22	0.23	0.815	1	212	211
	SSCC	0.93	0.49∼1.77	0.23	0.818	1	681	816
**GA vs GG**	overall	0.99	0.88∼1.12	0.08	0.933	7	2710	2786
	Caucasian	0.99	0.88∼1.12	0.08	0.933	7	2710	2786
	PB	0.96	0.84∼1.09	0.64	0.524	6	2204	2271
	SBCC	1.03	0.89∼1.20	0.41	0.680	5	1817	1759
	melanoma	0.68	0.43∼1.06	1.72	0.086	1	212	211
	SSCC	1.01	0.80∼1.27	0.10	0.923	1	681	816
**GA+AA vs GG**	overall	1.00	0.89∼1.13	0.07	0.620	7	2710	2786
	Caucasian	1.00	0.89∼1.13	0.07	0.620	6	2710	2786
	PB	0.95	0.84∼1.08	0.75	0.453	5	2204	2271
	SBCC	1.05	0.91∼1.21	0.63	0.527	1	1817	1759
	melanoma	0.71	0.47∼1.08	1.62	0.105	1	212	211
	SSCC	1.00	0.80∼1.25	0.03	0.974	7	681	816
**AA vs GG+GA**	overall	1.10	0.79∼1.55	0.57	0.569	6	2660	2726
	Caucasian	1.10	0.79∼1.55	0.57	0.569	6	2660	2726
	PB	0.90	0.62∼1.31	0.54	0.587	5	2154	2211
	SBCC	1.23	0.79∼1.93	0.92	0.359	4	1767	1699
	melanoma	1.00	0.41∼2.44	0.01	0.991	1	212	211
	SSCC	0.92	0.49∼1.75	0.24	0.809	1	681	816
**carrier A vs G**	overall	1.01	0.90∼1.12	0.10	0.920	7	2710	2786
	Caucasian	1.01	0.90∼1.12	0.10	0.920	7	2710	2786
	PB	0.96	0.85∼1.09	0.59	0.553	6	2204	2271
	SBCC	1.01	0.90∼1.19	0.53	0.595	5	1817	1759
	melanoma	0.78	0.53∼1.17	1.18	0.236	1	212	211
	SSCC	1.00	0.81∼1.24	0.01	0.996	1	681	816

SBCC: skin basal cell carcinomas; SSCC: skin squamous cell carcinoma, Ors: odd ratios; CIs: confidence intervals.

### Heterogeneity, publication bias and sensitivity analysis

Regarding the rs1800629 polymorphism and SCC risk, A vs G (I^2^ value of 77.5 % and *P*_heterogeneity_ <0.001), GA vs GG (I^2^=66.3 % and *P*_heterogeneity_ <0.001), GA+AA vs GG (I^2^=71.4 % and *P*_heterogeneity_ <0.001) and carrier A vs G (I^2^ =64.0 % and *P*_heterogeneity_ <0.001) data indicated a high degree of heterogeneity among the studies (Table 6). Thus, random-effect model was applied. In addition, fixed model was used in AA vs GG (I^2^=30.0 % and *P*_heterogeneity_=0.137) and AA vs GG+GA contrast (I^2^ =23.2 % and *P*_heterogeneity_ =0.203, Table 6).

**Table 6:** The analysis of heterogeneity and publication bias

Group	SNP	Comparison	Heterogeneity		Model	Begg's test		Egger's test	
			I^2^	*P_heterogeneity_*		z	*P_Begg_*	t	*P_Egger_*
**SSC**	rs1800629	A vs G	77.5%	<0.001	Random	0.49	0.621	0.07	0.942
		AA vs GG	30.0%	0.137	Fixed	1.20	0.228	1.67	0.120
		GA vs GG	66.3%	<0.001	Random	0.00	1.000	−0.42	0.683
		GA+AA vs GG	71.4%	<0.001	Random	−0.05	1.000	−0.12	0.903
		AA vs GG+GA	23.2%	0.203	Fixed	1.20	0.228	1.75	0.105
		carrier A vs G	64.0 %	<0.001	Random	0.00	1.000	−0.17	0.864
**SSC**	rs361525	A vs G	66.2%	0.019	Random	0.73	0.462	0.05	0.962
		GA vs GG	67.7%	0.015	Random	0.73	0.462	0.05	0.962
		GA+AA vs GG	59.8%	0.029	Random	0.38	0.707	0.09	0.930
		carrier A vs G	63.2%	0.028	Random	0.73	0.462	−0.03	0.978
**skin cancer**	rs1800629	A vs G	46.1%	0.084	Fixed	0.30	0.764	−0.68	0.528
		AA vs GG	38.6%	0.148	Fixed	0.38	0.707	0.33	0.756
		GA vs GG	45.4%	0.089	Fixed	0.30	0.764	−1.36	0.233
		GA+AA vs GG	46.6%	0.081	Fixed	0.60	0.548	−1.04	0.347
		AA vs GG+GA	38.1%	0.152	Fixed	0.38	0.707	0.49	0.650
		carrier A vs G	7.4%	0.372	Fixed	0.30	0.764	−0.95	0.387

SCC: squamous cell carcinoma; SNP: single nucleotide polymorphisms.

For the rs361525 polymorphism and SCC risk, random-effect model was used for the overall SCC, due to the presence of overall significant heterogeneity (Table 6, all I^2^>50 %, *P*_heterogeneity_<0.05). For the rs1800629 polymorphism and the risk of skin cancer, fixed-effect model was used for all models (Table 6, all I^2^ < 50 %, *P*_heterogeneity_ >0.05).

We also performed Begg's and Egger's tests to evaluate the potential publication bias among the included articles. The results indicate that publication bias can be ruled out for all comparisons (Table 6 and supplementary Figures 3-4; all *P*_Begg_>0.05, *P*_Egger_>0.05). Moreover, we conducted a sensitivity analysis and confirmed the stability of our results (Supplementary Figure 5 for rs1800629 and SCC risk; data no shown for others).

## DISCUSSION

In the present study, 16 case-control studies of *TNF-α* rs1800629 polymorphism [1, 5–9, 14, 16–24] and 6 case-control studies of rs361525 polymorphism [7, 8, 14, 20, 21, 24] were included in the meta-analysis of *TNF-α* polymorphism and the risk of SCC disease. We found that an increased overall SCC risk was associated with the rs1800629 polymorphisms in the Asian population under AA vs GG, and AA vs GG+GA models, but not A vs G, GA vs GG, GA+AA vs GG, or carrier A vs G models. A significant difference between LSCC/OSCC risks and the rs1800629 polymorphism was found under the AA vs GG, and AA vs GG+GA models; this corresponds with previous data on the link of rs1800629 and the risk of upper aero-digestive tract or head/neck SCC [25, 26]. However, in 2013, Chen et al performed a meta-analysis to analyze the association between rs1800629 and oral cancer, and observed a negative association between rs1800629 and OSCC [27]. Different selection criteria may contribute to this discrepancy. In our meta-analysis, two studies were excluded due to the requirement of Hardy-Weinberg equilibrium (HWE) or genotype data [28, 29]. Regarding the ESCC risk and rs1800629 polymorphism, the negative result was found under all genetic models, which was in line with the data of Luo et al [30]. The rs361525 allele was reported to be significantly increased in healthy controls compared with cancer patients, indicating a protective function [31]. Here, no significant difference was detected for rs361525 and overall SCC risks under all genetic models, which was partly in accordance with the data of Gao et al regarding head and neck SCC [26] and Zhou et al for overall cancer [32]. In addition, seven case-control studies in Caucasian population were included for the analysis of skin cancer [11–13, 15–17]. We failed to observe a significant association between *TNF-α* rs1800629 and skin cancer. In 2011, Nan et al also did not find any association between *TNF-α* gene variants and skin BCC or SCC in the Genome-Wide Association Studies (GWAS) from 2045 cases and 6013 controls of European population [33].

Although our results were validated by Begg's and Egger's tests, and by sensitivity analysis, the limitations in our meta-analysis should also be addressed. (1) Due to the limited number of studies published to date, only the common genetic polymorphisms of *TNF-α*, including rs1800629 and rs361525, were chosen. In addition, small sample size and/or limited genotype data in eligible articles affected our analysis. For example, there are two case-control studies of the association between *TNF-α* rs1800629 and melanoma risk [15, 34]. However, one study was excluded due to the departure of HWE [34]. The frequency data of GA+AA combined genotype and GG genotype were extracted in one OSCC study [24]. (2) A considerable heterogeneity was observed in the meta-analysis of rs1800629/rs361525 and the SSC risks. SCC has many different etiologies, and stratified analyses by every SCC disease type were not performed. The variations of clinical characteristics, ethnicity, geographical location, habits, gender, age and population feature were not fully considered. In spite of the use of random-effect model, a limited number of studies was included in the subgroup analysis. For example, only one case-control study was included for the rs1800629 and the susceptibility to a specific SCC disease, including SSCC [16] and OPSCC [21]. The subgroup analysis of LSCC and rs361525 was based on only 2 case-control studies [14, 20], and showed a positive correlation under A vs G model, GA vs GG model; GA+AA vs GG, and carrier A vs G model. It is possible that the GA genotype of rs361525 is associated with the decreased risk of LSCC. However, well-powered studies and stratified analyses by more factors are required to confirm our findings.

TNF-α is an important multifunctional pro-inflammatory cytokine, which is closely linked to the occurrence, progression, metastasis, prevention and therapy of many types of human cancer [35–37]. Alterations in *TNF-α* gene expression or TNF-α cytokine release lead to a variety of cancers [2, 38]. Genetic variation has been considered as a disease susceptibility or resistance factor [2]. The rs1800629 G/A polymorphism, located in the promoter region (−308 site) of human *TNF-α* gene, can lead to the substitution from G common allele to A rare allele [2]. *In vitro* experiments showed that the “A” rare allele of rs1800629 could increase *TNF-α* transcription [39, 40]. The frequency of “A” allele also positively correlates with high TNF-α levels in patients with oral cancer [28]. *TNF-α* rs1800629 was found to be positively associated with distant metastases of triple negative breast cancer patients [36]. However, no association was found between rs1800629 and *TNF-α* gene expression in gastric cancer patients [41, 42]. Here, we observed a positive correlation between the AA genotype of rs1800629 and the risks of LSCC/OSCC. However, we did not find any significant association between the A allele and the SCC risks. It is possible that the “A” rare allele functions in an allele dosage-dependent manner. TNF-α was found to increase the efficiency of chemotherapy and radiotherapy against breast cancer cells [43]. The carriage of the A rare allele of rs1800629 may be involved in this process, through inducing TNF-α transcription and protein expression. It may be meaningful to analyze the effect of combined mutations of *TNF-α* and other genes, including TNF-beta and interleukin-6, on the carcinogenesis and SCC cancer therapy, since this may lead to the discovery of potential novel biomarkers for SCC.

In conclusion, our meta-analysis indicates that the AA genotype of *TNF-α* rs1800629 polymorphism may serve as a prognostic biomarker for SCC, especially for LSCC and OSCC in the Asian population. The rs361525 polymorphism does not seem to be a genetic risk factor for SCC. In conjunction with other studies, these results provide a scientific support for the prognostic value of *TNF-α* rs1800629 polymorphisms in predicting the SCC risk.

## MATERIALS AND METHODS

### Database retrieval

The related articles published before January 17^th^, 2017 were searched in the electronic databases, including PUBMED, WOS, EMBASE, WANFANG, CNKI and SCOPUS, without any language restrictions. The present meta-analysis followed “Preferred Reporting Items for Systematic Reviews and Meta-Analyses” (PRISMA) [44], as shown in Supplementary Table 4.

### Article selection

Duplicated articles were removed by EndNote X7 software (Thomson Reuters). The following articles were excluded: 1) Reviews, theses, cases and trials; 2) Meeting abstracts and posters; 3) Cell or animal studies; 4) Other genes or diseases; 5) Meta-analyses; 6) Non-mutation data; 7) Data without detailed genotype; 8) Lack of control data; 9) Studies without specific oral cancer type information; 10) *P* value for HWE (*P*_HWE_) was less than 0.05; 11) Studies with unselected mutation sites. The selected articles provide sufficient information regarding the genotypes for *TNF-α* polymorphisms in case and control groups. *P*_HWE_ values were obtained by the chi-squared test.

### Data extraction and quality assessment

The authors extracted independently the following information: First author, publication year, country, ethnicity, number of cases/controls, source of controls, age (mean value), genotyping assay, gender (male %), SNP, genotype frequencies, disease type, χ^2^ and *P*_HWE_ values in control group. Newcastle-Ottawa Scale (NOS) system (http://www.ohri.ca/programs/clinical_epidemiology/oxford.asp) was used to assess the quality of the included studies; NOS score ≥7 indicates a high quality study.

### Statistical analyses

Mantel-Haenszel statistics was used to estimate the values of pooled odd radios (ORs) and 95 % confidence intervals (CIs); *P*_association_ value less than 0.05 was considered statistically significant. Six genetic models, including allele, homozygote, heterozygote, dominant recessive, or carrier models were used. Cochran Q statistic and I^2^ test were carried out to assess the potential heterogeneities between studies. When *P*_heterogeneity_ value of Cochran Q statistic > 0.05 or I^2^ value <50 %, the fixed-effect model was used. Otherwise, random-effect model was applied. To investigate the potential sources of heterogeneity, sensitivity analyses and subgroup analyses based on SCC disease type, ethnicity or source of controls were performed. Begg's test with pseudo 95 % confidence limits and Egger's test were also conducted to evaluate the potential publication bias. Stata/SE 12.0 (College Station, TX, USA) software was used for all statistical analyses.

## SUPPLEMENTARY FIGURES AND TABLES




