# Supplementary material for: Genetic association between TNF-α promoter polymorphism and susceptibility to squamous cell carcinoma, basal cell carcinoma, and melanoma: A meta-analysis
Source: Oncotarget. 2017 Apr 18;8(32):53873–85. doi: 10.18632/oncotarget.17179 (PMC5581156; doi:10.18632/oncotarget.17179)
Supplement: Supplementary file 1 [file oncotarget-08-53873-s001.pdf]

Genetic association between *TNF-α* promoter polymorphism and susceptibility to squamous cell carcinoma, basal cell carcinoma, and melanoma: A meta-analysis

SUPPLEMENTARY FIGURES AND TABLES

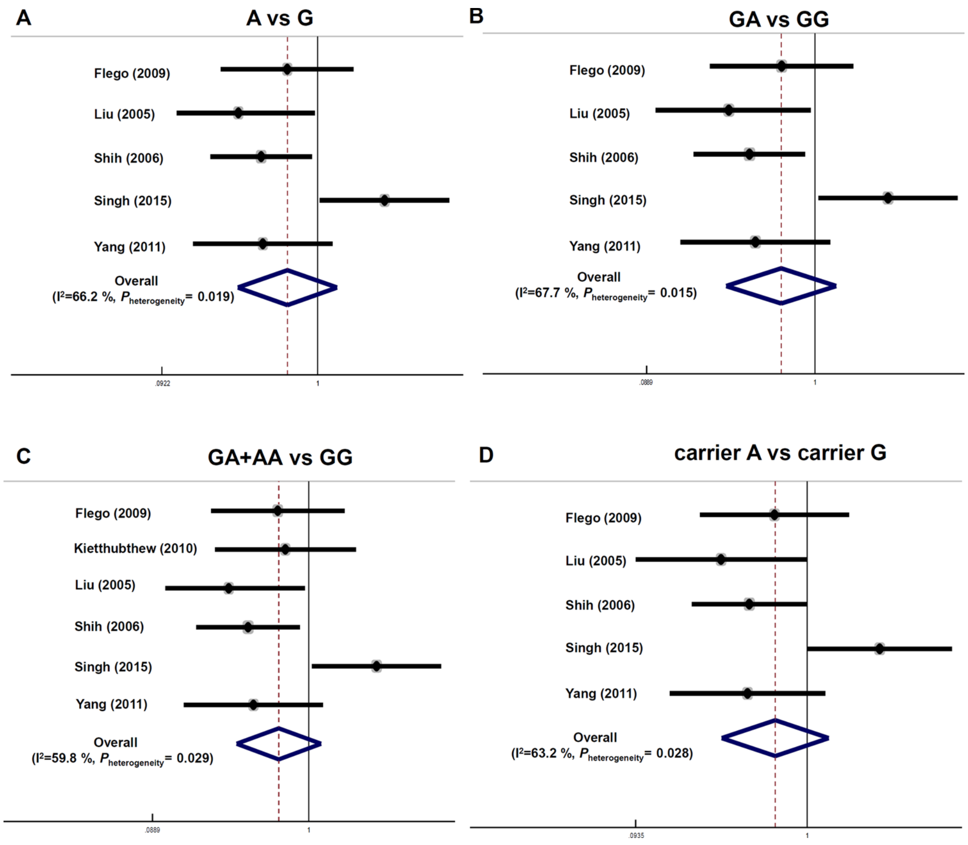

Supplementary Figure 1: Forest plot for the association between *TNF-α* rs361525 polymorphism and the risk of SCC. **(A)** A vs G model; **(B)** GA vs GG model; **(C)** GA+AA vs GG model; **(D)** carrier A vs carrier G.

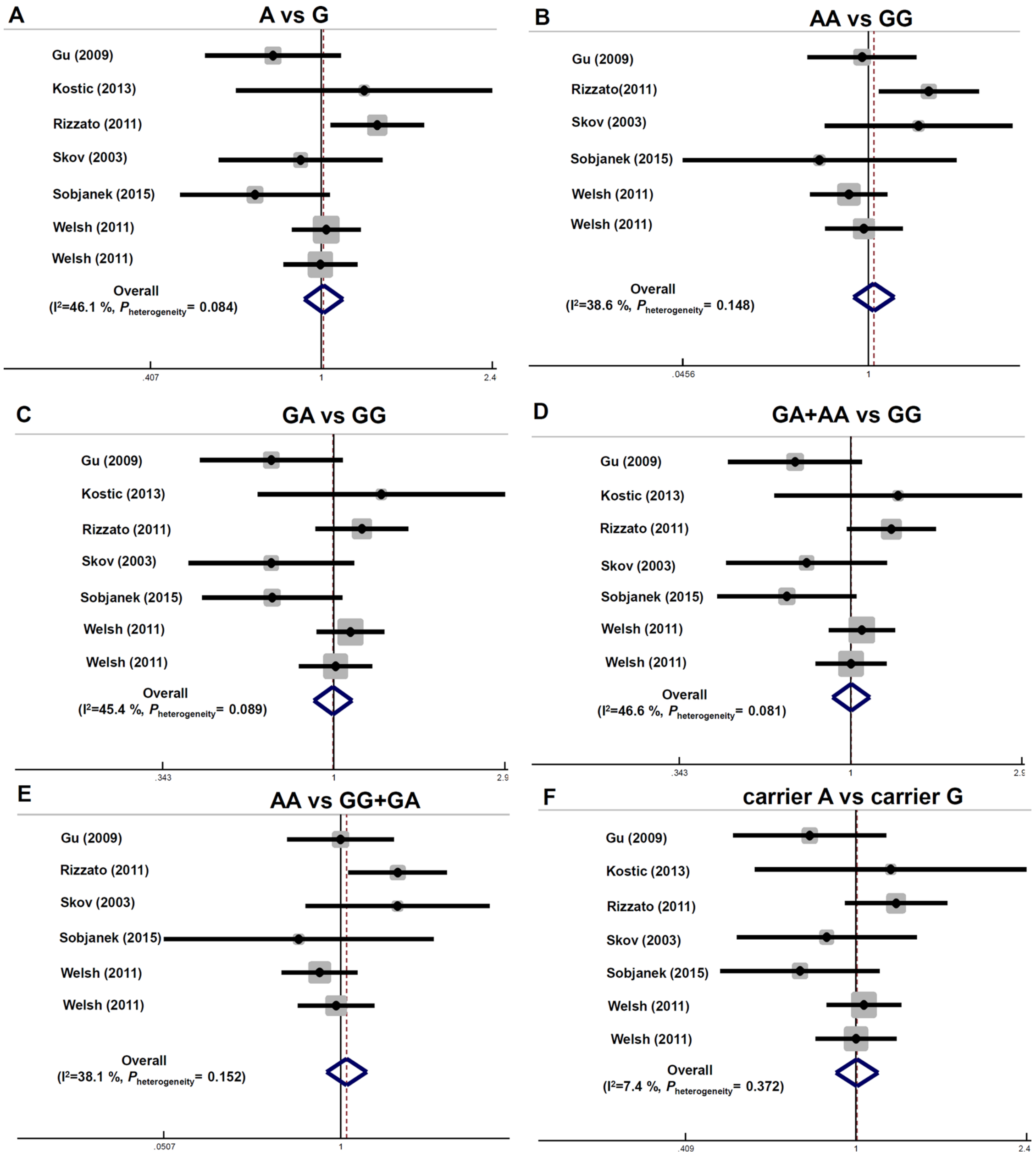

Supplementary Figure 2: Forest plot for the association between *TNF-α* rs1800629 polymorphism and the risk of skin cancer. **(A)** A vs G model; **(B)** AA vs GG model; **(C)** GA vs GG model; **(D)** GA+AA vs GG model; **(E)** AA vs GG+GA model; **(F)** carrier A vs carrier G model.

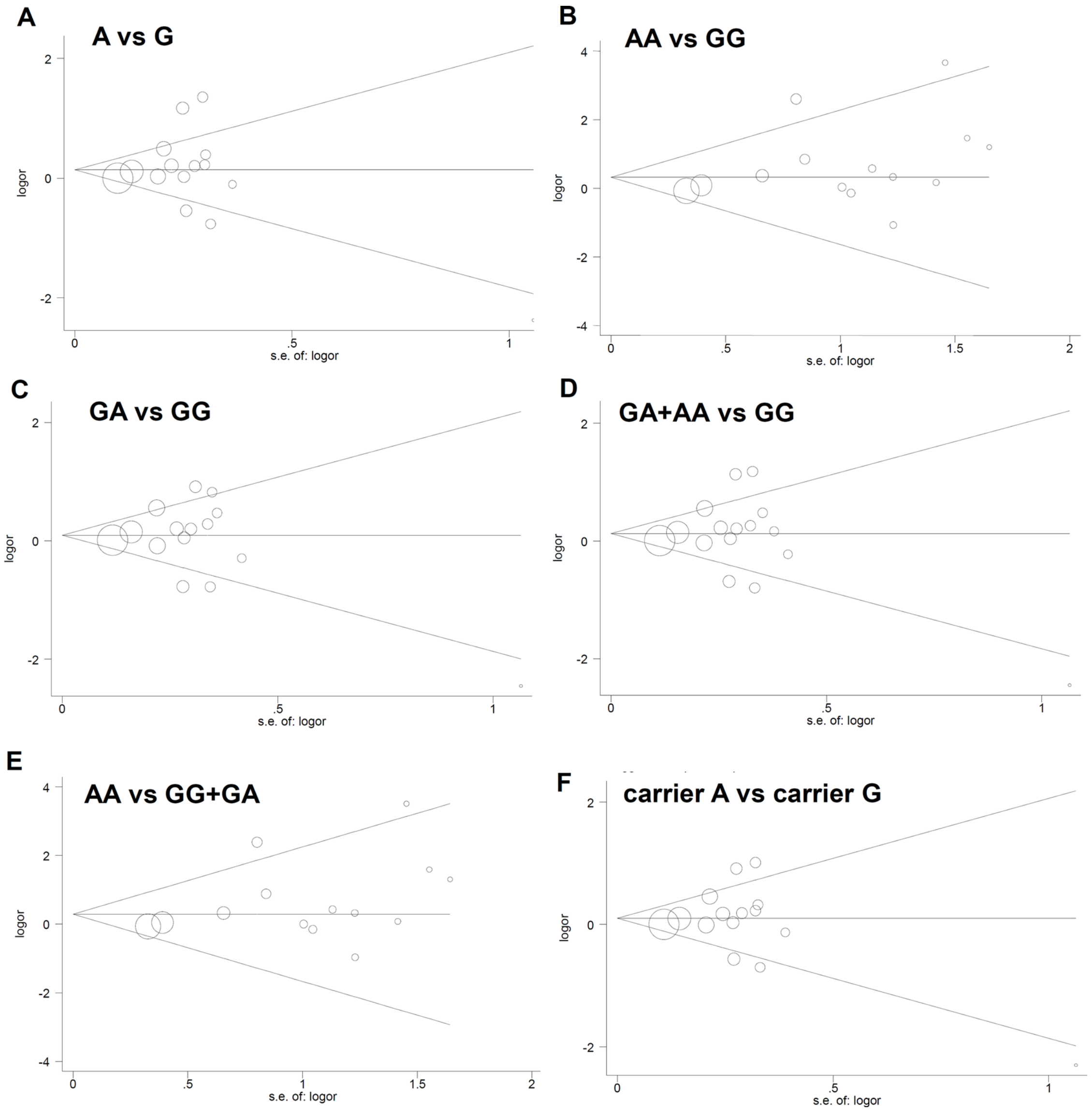

Supplementary Figure 3: Begg's funnel plot of publication bias for the association between *TNF-α* rs1800629 polymorphism and the risk of SCC. **(A)** A vs G model; **(B)** AA vs GG model; **(C)** GA vs GG model; **(D)** GA+AA vs GG model; **(E)** AA vs GG+GA model; **(F)** carrier A vs carrier G model.

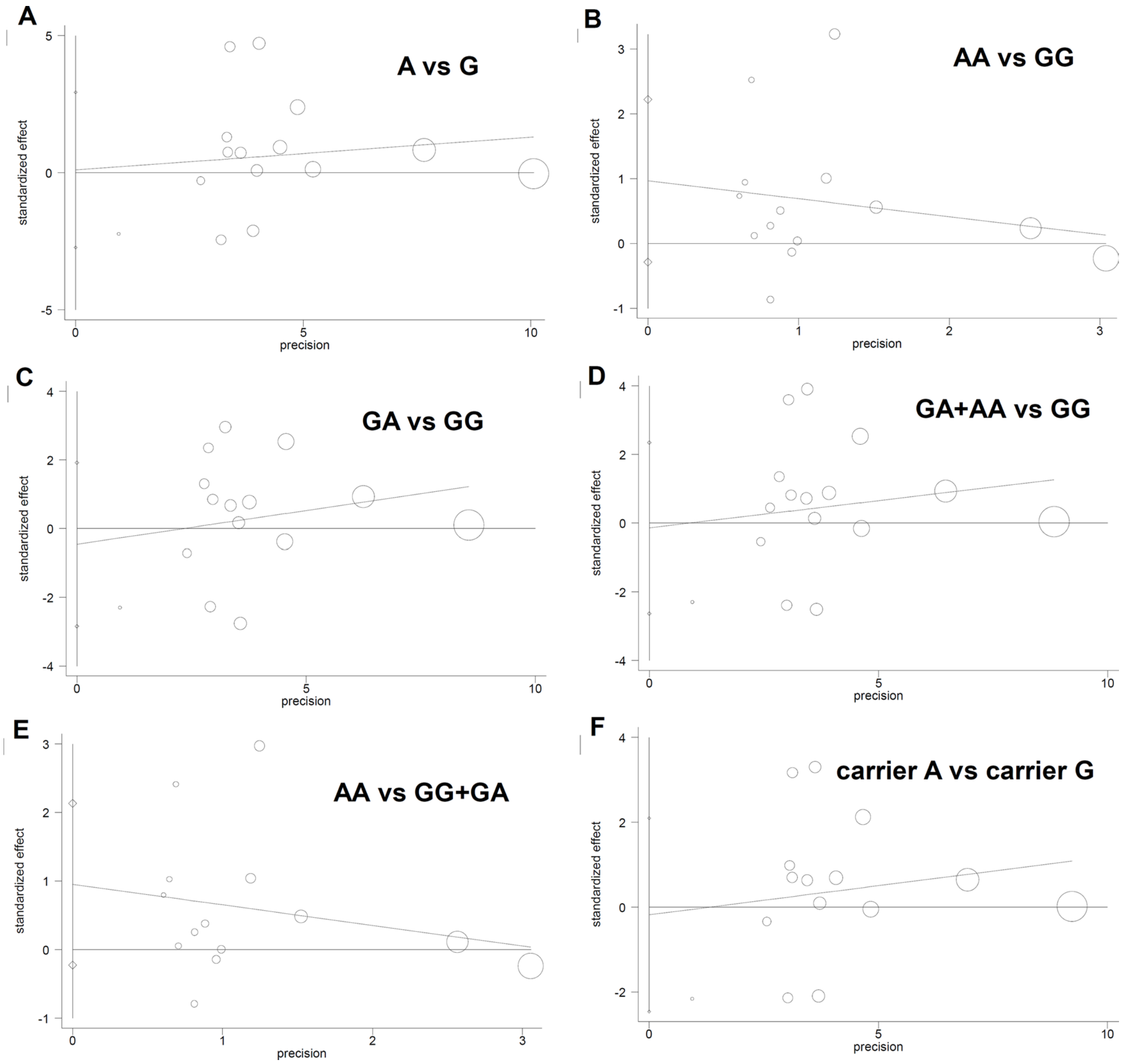

Supplementary Figure 4: Egger's test for the association between *TNF-α* rs1800629 polymorphism and the risk of SCC. **(A)** A vs G model; **(B)** AA vs GG model; **(C)** GA vs GG model; **(D)** GA+AA vs GG model; **(E)** AA vs GG+GA model; **(F)** carrier A vs carrier G model.

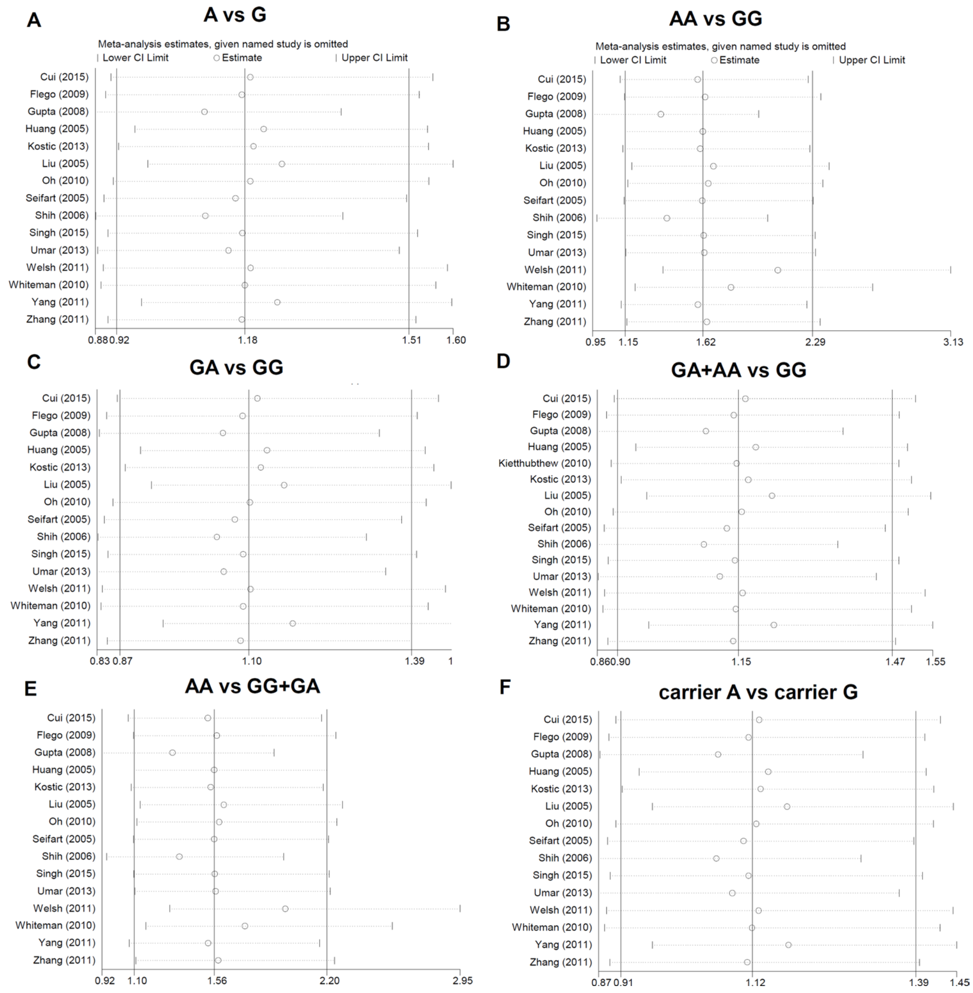

Supplementary Figure 5: The sensitivity meta-analysis for the association between *TNF-α* rs1800629 polymorphism and the risk of SCC. **(A)** A vs G model; **(B)** AA vs GG model; **(C)** GA vs GG model; **(D)** GA+AA vs GG model; **(E)** AA vs GG+GA model; **(F)** carrier A vs carrier G model.

Supplementary Table 1: The detailed search strategy

See Supplementary File 1

Supplementary Table 2: The excluded full-text articles

See Supplementary File 1

Supplementary Table 3: Quality assessment of the included studies according to the Newcastle-Ottawa Scale (NOS)

See Supplementary File 1

Supplementary Table 4: Meta-analysis on Genetic Association Studies Checklist

See Supplementary File 1
