# Supplementary material for: Genetic association between TNF-α promoter polymorphism and susceptibility to squamous cell carcinoma, basal cell carcinoma, and melanoma: A meta-analysis
Source: Oncotarget. 2017 Apr 18;8(32):53873–85. doi: 10.18632/oncotarget.17179 (PMC5581156; doi:10.18632/oncotarget.17179)
Supplement: Supplementary file 2 [file oncotarget-08-53873-s002.doc]

# Supplementary Table S1: The detailed search strategy

| 1. **PUBMED database** |
| --- |
| **Step 1: (#1) Number: 223817**  (((((((((Tumor Necrosis Factor-alpha[MeSH Terms]) OR Tumor Necrosis Factor alpha) OR Cachectin-Tumor Necrosis Factor) OR Cachectin Tumor Necrosis Factor) OR TNFalpha) OR TNF-alpha) OR Tumor Necrosis Factor) OR Tumor Necrosis Factor Ligand Superfamily Member 2) OR Cachectin) OR TNF Superfamily, Member 2  **Step 2: (#2) Number: 276827**  ((((Polymorphism, Genetic[MeSH Terms]) OR Polymorphisms, Genetic) OR Genetic Polymorphism) OR Polymorphism (Genetics)) OR Genetic Polymorphisms  **Step 3: (#3) Number: 147563**  (((((((((((((((Carcinoma, Squamous Cell[MeSH Terms]) OR Carcinomas, Squamous Cell) OR Squamous Cell Carcinomas) OR Squamous Cell Carcinoma) OR Carcinoma, Squamous) OR Carcinomas, Squamous) OR Squamous Carcinoma) OR Squamous Carcinomas) OR Carcinoma, Epidermoid) OR Carcinomas, Epidermoid) OR Epidermoid Carcinoma) OR Epidermoid Carcinomas) OR Carcinoma, Planocellular) OR Carcinomas, Planocellular) OR Planocellular Carcinoma) OR Planocellular Carcinomas  **Step 4: (#4) Number: 26074**  ((((((((((((Carcinoma, Basal Cell[MeSH Terms]) OR Basal Cell Carcinoma) OR Basal Cell Carcinomas) OR Carcinomas, Basal Cell) OR Rodent Ulcer) OR Rodent Ulcers) OR Ulcers, Rodent) OR Ulcer, Rodent) OR Epithelioma, Basal Cell) OR Basal Cell Epithelioma) OR Basal Cell Epitheliomas) OR Epitheliomas, Basal Cell) OR Carcinoma, Basal Cell, Pigmented  **Step 5: (#5) Number: 111544**  ((((Melanomas[MeSH Terms]) OR Malignant Melanoma) OR Malignant Melanomas) OR Melanoma, Malignant) OR Melanomas, Malignant  **Step 6: (#1 And #2= #6) Number: 6234**  (((((((((((Tumor Necrosis Factor-alpha[MeSH Terms]) OR Tumor Necrosis Factor alpha) OR Cachectin-Tumor Necrosis Factor) OR Cachectin Tumor Necrosis Factor) OR TNFalpha) OR TNF-alpha) OR Tumor Necrosis Factor) OR Tumor Necrosis Factor Ligand Superfamily Member 2) OR Cachectin) OR TNF Superfamily, Member 2)) AND (((((Polymorphism, Genetic[MeSH Terms]) OR Polymorphisms, Genetic) OR Genetic Polymorphism) OR Polymorphism (Genetics)) OR Genetic Polymorphisms)  **Step 7: (#3 OR #4 OR #5= #7) Number: 268639**  ((((((((((((((((((Carcinoma, Squamous Cell[MeSH Terms]) OR Carcinomas, Squamous Cell) OR Squamous Cell Carcinomas) OR Squamous Cell Carcinoma) OR Carcinoma, Squamous) OR Carcinomas, Squamous) OR Squamous Carcinoma) OR Squamous Carcinomas) OR Carcinoma, Epidermoid) OR Carcinomas, Epidermoid) OR Epidermoid Carcinoma) OR Epidermoid Carcinomas) OR Carcinoma, Planocellular) OR Carcinomas, Planocellular) OR Planocellular Carcinoma) OR Planocellular Carcinomas)) OR (((((((((((((Carcinoma, Basal Cell[MeSH Terms]) OR Basal Cell Carcinoma) OR Basal Cell Carcinomas) OR Carcinomas, Basal Cell) OR Rodent Ulcer) OR Rodent Ulcers) OR Ulcers, Rodent) OR Ulcer, Rodent) OR Epithelioma, Basal Cell) OR Basal Cell Epithelioma) OR Basal Cell Epitheliomas) OR Epitheliomas, Basal Cell) OR Carcinoma, Basal Cell, Pigmented)) OR (((((Melanomas[MeSH Terms]) OR Malignant Melanoma) OR Malignant Melanomas) OR Melanoma, Malignant) OR Melanomas, Malignant)  **Step 8: (#6 And #7) Number: 94**  (((((((((((((Tumor Necrosis Factor-alpha[MeSH Terms]) OR Tumor Necrosis Factor alpha) OR Cachectin-Tumor Necrosis Factor) OR Cachectin Tumor Necrosis Factor) OR TNFalpha) OR TNF-alpha) OR Tumor Necrosis Factor) OR Tumor Necrosis Factor Ligand Superfamily Member 2) OR Cachectin) OR TNF Superfamily, Member 2)) AND (((((Polymorphism, Genetic[MeSH Terms]) OR Polymorphisms, Genetic) OR Genetic Polymorphism) OR Polymorphism (Genetics)) OR Genetic Polymorphisms))) AND (((((((((((((((((((Carcinoma, Squamous Cell[MeSH Terms]) OR Carcinomas, Squamous Cell) OR Squamous Cell Carcinomas) OR Squamous Cell Carcinoma) OR Carcinoma, Squamous) OR Carcinomas, Squamous) OR Squamous Carcinoma) OR Squamous Carcinomas) OR Carcinoma, Epidermoid) OR Carcinomas, Epidermoid) OR Epidermoid Carcinoma) OR Epidermoid Carcinomas) OR Carcinoma, Planocellular) OR Carcinomas, Planocellular) OR Planocellular Carcinoma) OR Planocellular Carcinomas)) OR (((((((((((((Carcinoma, Basal Cell[MeSH Terms]) OR Basal Cell Carcinoma) OR Basal Cell Carcinomas) OR Carcinomas, Basal Cell) OR Rodent Ulcer) OR Rodent Ulcers) OR Ulcers, Rodent) OR Ulcer, Rodent) OR Epithelioma, Basal Cell) OR Basal Cell Epithelioma) OR Basal Cell Epitheliomas) OR Epitheliomas, Basal Cell) OR Carcinoma, Basal Cell, Pigmented)) OR (((((Melanomas[MeSH Terms]) OR Malignant Melanoma) OR Malignant Melanomas) OR Melanoma, Malignant) OR Melanomas, Malignant)) |
| 1. **WOS database** |
| ((TOPIC: ((((((Tumor Necrosis Factor-alpha OR cachectic Tumor Necrosis Factor) OR TNFalpha) OR TNF-alpha) OR Tumor Necrosis Factor Ligand Superfamily Member 2) OR TNF Superfamily, Member 2) OR TNF-α) AND TOPIC: (((((polymorphism OR polymorphisms) OR mutation) OR mutations) OR SNP) OR Single Nucleotide Polymorphism)) AND TOPIC: (((((((squamous cell carcinoma OR SCC) OR BCC) OR Basal cell carcinoma) OR Melanoma) OR Melanomas) OR Malignant Melanoma) OR Malignant Melanomas))  Timespan: All years.  Search language=Auto  **Number: 388** |
| 1. **EMBASE database** |
| **Step 1: (#1), Number: 145625**  'tumor necrosis factor-alpha' OR 'cachectin tumor necrosis factor' OR 'tnfalpha' OR 'tnf-alpha' OR 'tumor necrosis factor ligand superfamily member 2' OR 'tnf superfamily, member 2' OR 'tnf-α'  **Step 2: (#2), Number: 1151380**  'polymorphism' OR 'polymorphisms' OR 'mutation' OR 'mutations' OR 'snp' OR 'single nucleotide polymorphism'  **Step 3: (#3), Number: 332744**  'squamous cell carcinoma' OR 'scc' OR 'bcc' OR 'basal cell carcinoma' OR 'melanoma' OR 'melanomas' OR 'malignant melanoma' OR 'malignant melanomas'  **Step 4: (#1 And #2 And #3), Number: 144**  'tumor necrosis factor-alpha' OR 'cachectin tumor necrosis factor' OR 'tnfalpha' OR 'tnf-alpha' OR 'tumor necrosis factor ligand superfamily member 2' OR 'tnf superfamily, member 2' OR 'tnf-α' AND ('polymorphism' OR 'polymorphisms' OR 'mutation' OR 'mutations' OR 'snp' OR 'single nucleotide polymorphism') AND ('squamous cell carcinoma' OR 'scc' OR 'bcc' OR 'basal cell carcinoma' OR 'melanoma' OR 'melanomas' OR 'malignant melanoma' OR 'malignant melanomas') |
| 1. **WANFANG database** |
| TNF-α * 鳞状细胞癌 + 基底细胞癌 + 黑色素瘤 * 基因多态性 * Date:-2017 * Date:1990-2017  **Number: 388** |
| 1. **CNKI database** |
| **Step 1: Number: 10**  主题=中英文扩展(TNF-α and 主题=中英文扩展(鳞状细胞癌 and 主题=中英文扩展(基因多态性 and (模糊匹配)  **Step 2: Number: 0**  主题=中英文扩展(TNF-α and 主题=中英文扩展(基底细胞癌and 主题=中英文扩展(基因多态性 and (模糊匹配)  **Step 3: Number: 1**  主题=中英文扩展(TNF-α and 主题=中英文扩展(黑色素瘤 and 主题=中英文扩展(基因多态性 and (模糊匹配) |
| 1. **SCOPUS database** |
| (TITLE-ABS-KEY("Tumor Necrosis Factor-alpha" OR "Cachectin Tumor Necrosis Factor" OR "TNFalpha" OR "TNF-alpha" OR "Tumor Necrosis Factor Ligand Superfamily Member 2" OR "TNF Superfamily, Member 2" OR "TNF-α") AND TITLE-ABS-KEY ("polymorphism" OR "polymorphisms" OR "mutation" OR "mutations" OR "SNP" OR "Single Nucleotide Polymorphism") AND TITLE-ABS-KEY ("squamous cell carcinoma" OR "SCC" OR "BCC" OR "Basal cell carcinoma" OR "Melanoma" OR "Melanomas" OR "Malignant Melanoma" OR "Malignant Melanomas"))  **Number: 304** |

# Supplementary Table S2: The excluded full-text articles

| **Cause 1^st^: Data without detailed genotype** |
| --- |
| **Number: 5**   1. Pacholczyk M, Czernicki J and Ferenc T. [The effect of solar ultraviolet radiation (UVR) on induction of skin cancers]. Medycyna pracy. 2016; 67(2):255-266. 2. Roszak A, Misztal M, Sowinska A and Jagodzinski PP. TNF-alpha -308 G/A as a risk marker of cervical cancer progression in the Polish population. Molecular diagnosis & therapy. 2015; 19(1):53-57. 3. Bodelon C, Madeleine MM, Johnson LG, Du Q, Galloway DA, Malkki M, Petersdorf EW and Schwartz SM. Genetic variation in the TLR and NF-kappaB pathways and cervical and vulvar cancer risk: a population-based case-control study. International journal of cancer Journal international du cancer. 2014; 134(2):437-444. 4. Golozar A, Beaty TH, Gravitt PE, Ruczinski I, Qiao YL, Fan JH, Ding T, Tang ZZ, Etemadi A, Hu N, Hyland PL, Wang L, Wang C, Dawsey SM, Freedman ND, Abnet CC, et al. Oesophageal squamous cell carcinoma in high-risk Chinese populations: Possible role for vascular epithelial growth factor A. European journal of cancer (Oxford, England : 1990). 2014; 50(16):2855-2865. 5. Jin L, Sturgis EM, Zhang Y, Huang Z, Song X, Li C, Wei Q and Li G. Association of tumor necrosis factor-alpha promoter variants with risk of HPV-associated oral squamous cell carcinoma. Molecular cancer. 2013; 12:80. |
| **Cause 2^nd^: Lack of control data** |
| **Number: 7**   1. Zhang P, Seth A, Baisre A and Fernandes H. Differential signaling patterns in HPV positive and negative HNSCC identified by next-generation sequencing. Journal of Molecular Diagnostics. 2013; 15(6):918. 2. Ramachandran S, Fryer AA, Smith AG, Lear JT, Bowers B, Hartland AJ, Whiteside JR, Jones PW and Strange RC. Basal cell carcinomas: association of allelic variants with a high-risk subgroup of patients with the multiple presentation phenotype. Pharmacogenetics. 2001; 11(3):247-254. 3. Zhang C, Sturgis EM, Zheng H, Song X, Wei P, Jin L, Chao L, Wei Q and Li G. Genetic variants in TNF-alpha promoter are predictors of recurrence in patients with squamous cell carcinoma of oropharynx after definitive radiotherapy. International journal of cancer Journal international du cancer. 2014; 134(8):1907-1915. 4. Erdei E, Luo L, Sheng H, Maestas E, White KA, Mackey A, Dong Y, Berwick M and Morse DE. Cytokines and tumor metastasis gene variants in oral cancer and precancer in Puerto Rico. PLoS One. 2013; 8(11):e79187. 5. Correa GT, Bandeira GA, Cavalcanti BG, de Carvalho Fraga CA, dos Santos EP, Silva TF, Gomez RS, Guimaraes AL and De Paula AM. Association of -308 TNF-alpha promoter polymorphism with clinical aggressiveness in patients with head and neck squamous cell carcinoma. Oral oncology. 2011; 47(9):888-894. 6. Zhang C, Sturgis EM, Zheng H, Zafereo ME, Wei Q and Li G. TNF-α promoter polymorphisms and risk of recurrence in patients with squamous cell carcinomas of the nonoropharynx. International Journal of Cancer. 2014; 135(7):1615-1624. 7. Omatsu H, Kuwahara A, Yamamori M, Fujita M, Okuno T, Miki I, Tamura T, Nishiguchi K, Okamura N, Nakamura T, Azuma T, Hirano T, Ozawa K and Hirai M. TNF-alpha -857C>T genotype is predictive of clinical response after treatment with definitive 5-fluorouracil/cisplatin-based chemoradiotherapy in Japanese patients with esophageal squamous cell carcinoma. International journal of medical sciences. 2013; 10(12):1755-1760. |
| **Cause 3^th^: Without specific oral cancer type information** |
| **Number: 2**   1. Chiu CJ, Chiang CP, Chang ML, Chen HM, Hahn LJ, Hsieh LL, Kuo YS and Chen CJ. Association between genetic polymorphism of tumor necrosis factor-alpha and risk of oral submucous fibrosis, a pre-cancerous condition of oral cancer. Journal of dental research. 2001; 80(12):2055-2059. 2. Chen WC, Tsai MH, Lei W, Chen WC, Tsai CH and Tsai FJ. CYP17 and tumor necrosis factor-alpha gene polymorphisms are associated with risk of oral cancer in Chinese patients in Taiwan. Acta oto-laryngologica. 2005; 125(1):96-99. |
| Cause 4^th^: ***P* value for HWE<0.05** |
| **Number: 4**   1. Howell WM, Turner SJ, Collins A, Bateman AC and Theaker JM. Influence of TNFalpha and LTalpha single nucleotide polymorphisms on susceptibility to and prognosis in cutaneous malignant melanoma in the British population. European journal of immunogenetics : official journal of the British Society for Histocompatibility and Immunogenetics. 2002; 29(1):17-23. 2. Vairaktaris E, Yapijakis C, Serefoglou Z, Avgoustidis D, Critselis E, Spyridonidou S, Vylliotis A, Derka S, Vassiliou S, Nkenke E and Patsouris E. Gene expression polymorphisms of interleukins-1 beta, -4, -6, -8, -10, and tumor necrosis factors-alpha, -beta: regression analysis of their effect upon oral squamous cell carcinoma. Journal of cancer research and clinical oncology. 2008; 134(8):821-832. 3. Yapijakis C, Serefoglou Z, Vylliotis A, Nkenke E, Derka S, Vassiliou S, Avgoustidis D, Neukam FW, Patsouris E and Vairaktaris E. Association of polymorphisms in Tumor Necrosis Factor Alpha and Beta genes with increased risk for oral cancer. Anticancer Res. 2009; 29(6):2379-2386. 4. Guo W, Wang N, Li Y and Zhang JH. Polymorphisms in tumor necrosis factor genes and susceptibility to esophageal squamous cell carcinoma and gastric cardiac adenocarcinoma in a population of high incidence region of North China. Chin Med J (Engl). 2005; 118(22):1870-1878. |
| Cause 5^th^: **Unselected mutation sites** |
| **Number: 7**   1. Jahnke V, Matthias C, Bockmuhl U and Strange RC. [Genetic predisposition for the development of head and neck carcinomas]. Laryngo- rhino- otologie. 1999; 78(1):24-27. 2. Matthias C, Jahnke V, Fryer A, Strange R, Ollier W and Hajeer A. Influence of tumour necrosis factor microsatellite polymorphisms on susceptibility to head and neck cancer. Acta oto-laryngologica. 1998; 118(2):284-288. 3. Matthias C, Jahnke V, Fryer AA and Strange RC. [First results on the influence of polymorphisms at glutathione S-transferase, cytochrome P450, and tumor necrosis factor gene loci on the development of multiple head and neck cancer]. Laryngo- rhino- otologie. 2003; 82(1):25-30. 4. Matthias C, Jahnke V, Hajeer A, Ollier W, Fryer AA and Strange RC. [Influence of genetic variation in the major histocompatibility complex on head and neck cancer susceptibility]. Laryngo- rhino- otologie. 2001; 80(10):595-600. 5. Hajeer AH, Lear JT, Ollier WE, Naves M, Worthington J, Bell DA, Smith AG, Bowers WP, Jones PW, Strange RC and Fryer AA. Preliminary evidence of an association of tumour necrosis factor microsatellites with increased risk of multiple basal cell carcinomas. The British journal of dermatology. 2000; 142(3):441-445. 6. Hardikar S, Johnson LG, Malkki M, Petersdorf EW, Galloway DA, Schwartz SM and Madeleine MM. A population-based case-control study of genetic variation in cytokine genes associated with risk of cervical and vulvar cancers. Gynecologic oncology. 2015; 139(1):90-96. 7. Yin G, Zhu T, Li J, Wu A, Liang J and Zhi Y. CXCL12 rs266085 and TNF-alpha rs1799724 polymorphisms and susceptibility to cervical cancer in a Chinese population. International journal of clinical and experimental pathology. 2015; 8(5):5768-5774. |

# Supplementary Table S3: Quality assessment of the included studies according to the Newcastle-Ottawa Scale (NOS)

| **Author**  **(Ref)** | **Year** | **Case** | | **Control** | | **Comparability** | | **Exposure** | | | | **NOS score** |
| --- | --- | --- | --- | --- | --- | --- | --- | --- | --- | --- | --- | --- |
|  |  | **Definition** | **Representativeness** | **Selection** | **Definition** | **Important factors** | **Other factors** | **Secure record** | **Blind** | **Method** | **Non-response rate** |  |
| **Cui** | 2015 | ★ | ★ | ★ | ★ | ★ | ★ | ☆ | ☆ | ★ | ★ | **8** |
| **Flego** | 2009 | ★ | ★ | ★ | ★ | ★ | ★ | ☆ | ☆ | ★ | ★ | **8** |
| **Gu** | 2009 | ☆ | ★ | ★ | ★ | ★ | ★ | ★ | ★ | ★ | ☆ | **8** |
| **Gupta** | 2008 | ★ | ★ | ★ | ★ | ★ | ★ | ☆ | ☆ | ★ | ★ | **8** |
| **Huang** | 2005 | ★ | ★ | ★ | ★ | ☆ | ★ | ☆ | ☆ | ★ | ★ | **7** |
| **Kietthubthew** | 2010 | ☆ | ★ | ★ | ★ | ★ | ☆ | ★ | ★ | ★ | ☆ | **7** |
| **Kostic** | 2013 | ☆ | ★ | ★ | ★ | ★ | ★ | ☆ | ☆ | ★ | ★ | **7** |
| **Liu** | 2005 | ☆ | ★ | ★ | ★ | ★ | ★ | ★ | ★ | ★ | ★ | **9** |
| **Oh** | 2010 | ☆ | ★ | ★ | ★ | ★ | ★ | ☆ | ☆ | ★ | ★ | **7** |
| **Rizzato** | 2011 | ★ | ★ | ☆ | ☆ | ★ | ★ | ★ | ★ | ★ | ☆ | **7** |
| **Seifart** | 2005 | ☆ | ★ | ★ | ★ | ★ | ☆ | ★ | ★ | ★ | ☆ | **7** |
| **Shih** | 2006 | ☆ | ★ | ★ | ★ | ★ | ★ | ★ | ★ | ★ | ★ | **9** |
| **Singh** | 2015 | ☆ | ★ | ★ | ★ | ★ | ★ | ☆ | ☆ | ★ | ★ | **7** |
| **Skov** | 2003 | ☆ | ★ | ★ | ★ | ★ | ☆ | ★ | ★ | ★ | ★ | **8** |
| **Sobjanek** | 2015 | ☆ | ★ | ★ | ★ | ★ | ★ | ☆ | ☆ | ★ | ★ | **7** |
| **Umar** | 2013 | ☆ | ★ | ★ | ★ | ★ | ★ | ★ | ★ | ★ | ☆ | **8** |
| **Welsh** | 2011 | ★ | ★ | ★ | ★ | ★ | ★ | ☆ | ☆ | ★ | ☆ | **7** |
| **Whiteman** | 2010 | ☆ | ★ | ★ | ★ | ★ | ☆ | ★ | ★ | ★ | ☆ | **7** |
| **Yang** | 2011 | ☆ | ★ | ★ | ★ | ★ | ★ | ★ | ★ | ★ | ★ | **9** |
| **Zhang** | 2011 | ★ | ★ | ☆ | ★ | ★ | ★ | ☆ | ☆ | ★ | ★ | **7** |

**★, score value=1; ☆, score value=0;The specific item information is available from http://www.ohri.ca/programs/clinical_epidemiology/oxford.asp.**

# Supplementary Table S4: Meta-analysis on Genetic Association Studies Checklist

| **Section/topic** | **#** | **Checklist item** | **Reported on page #** |
| --- | --- | --- | --- |
| **TITLE** | | |  |
| Title | 1 | Identify the report as a systematic review, meta-analysis, or both. | 1 |
| **ABSTRACT** | | |  |
| Structured summary | 2 | Provide a structured summary including, as applicable: background; objectives; data sources; study eligibility criteria, participants, and interventions; study appraisal and synthesis methods; results; limitations; conclusions and implications of key findings; systematic review registration number. | 2 |
| **INTRODUCTION** | | |  |
| Rationale | 3 | Describe the rationale for the review in the context of what is already known. | 3-4 |
| Objectives | 4 | Provide an explicit statement of questions being addressed with reference to participants, interventions, comparisons, outcomes, and study design (PICOS). | 3-4 |
| **METHODS** | | |  |
| Protocol and registration | 5 | Indicate if a review protocol exists, if and where it can be accessed (e.g., Web address), and, if available, provide registration information including registration number. | - |
| Eligibility criteria | 6 | Specify study characteristics (e.g., PICOS, length of follow-up) and report characteristics (e.g., years considered, language, publication status) used as criteria for eligibility, giving rationale. | 11-12 |
| Information sources | 7 | Describe all information sources (e.g., databases with dates of coverage, contact with study authors to identify additional studies) in the search and date last searched. | 11-12 |
| Search | 8 | Present full electronic search strategy for at least one database, including any limits used, such that it could be repeated. | 11 |
| Study selection | 9 | State the process for selecting studies (i.e., screening, eligibility, included in systematic review, and, if applicable, included in the meta-analysis). | 11 |
| Data collection process | 10 | Describe method of data extraction from reports (e.g., piloted forms, independently, in duplicate) and any processes for obtaining and confirming data from investigators. | 11 |
| Data items | 11 | List and define all variables for which data were sought (e.g., PICOS, funding sources) and any assumptions and simplifications made. | 11-12 |
| Risk of bias in individual studies | 12 | Describe methods used for assessing risk of bias of individual studies (including specification of whether this was done at the study or outcome level), and how this information is to be used in any data synthesis. | 12 |
| Summary measures | 13 | State the principal summary measures (e.g., risk ratio, difference in means). | 12 |
| Synthesis of results | 14 | Describe the methods of handling data and combining results of studies, if done, including measures of consistency (e.g., I^2^) for each meta-analysis. | 12 |

Page 1 of 2

| **Section/topic** | **#** | **Checklist item** | **Reported on page #** |
| --- | --- | --- | --- |
| Risk of bias across studies | 15 | Specify any assessment of risk of bias that may affect the cumulative evidence (e.g., publication bias, selective reporting within studies). | 12 |
| Additional analyses | 16 | Describe methods of additional analyses (e.g., sensitivity or subgroup analyses, meta-regression), if done, indicating which were pre-specified. | 12 |
| **RESULTS** | | |  |
| Study selection | 17 | Give numbers of studies screened, assessed for eligibility, and included in the review, with reasons for exclusions at each stage, ideally with a flow diagram. | 4 |
| Study characteristics | 18 | For each study, present characteristics for which data were extracted (e.g., study size, PICOS, follow-up period) and provide the citations. | 4 |
| Risk of bias within studies | 19 | Present data on risk of bias of each study and, if available, any outcome level assessment (see item 12). | 7 |
| Results of individual studies | 20 | For all outcomes considered (benefits or harms), present, for each study: (a) simple summary data for each intervention group (b) effect estimates and confidence intervals, ideally with a forest plot. | 5-7 |
| Synthesis of results | 21 | Present results of each meta-analysis done, including confidence intervals and measures of consistency. | 5-7 |
| Risk of bias across studies | 22 | Present results of any assessment of risk of bias across studies (see Item 15). | 7 |
| Additional analysis | 23 | Give results of additional analyses, if done (e.g., sensitivity or subgroup analyses, meta-regression [see Item 16]). | 5-7 |
| **DISCUSSION** | | |  |
| Summary of evidence | 24 | Summarize the main findings including the strength of evidence for each main outcome; consider their relevance to key groups (e.g., healthcare providers, users, and policy makers). | 7-11 |
| Limitations | 25 | Discuss limitations at study and outcome level (e.g., risk of bias), and at review-level (e.g., incomplete retrieval of identified research, reporting bias). | 9 |
| Conclusions | 26 | Provide a general interpretation of the results in the context of other evidence, and implications for future research. | 10-11 |
| **FUNDING** | | |  |
| Funding | 27 | Describe sources of funding for the systematic review and other support (e.g., supply of data); role of funders for the systematic review. | - |

*From:*  Moher D, Liberati A, Tetzlaff J, Altman DG, The PRISMA Group (2009). Preferred Reporting Items for Systematic Reviews and Meta-Analyses: The PRISMA Statement. PLoS Med 6(6): e1000097. doi:10.1371/journal.pmed1000097 For more information, visit: **www.prisma-statement.org**.

Page 2 of 2
